# A prognostic score based on B cell and plasma cell densities compared to T cell densities in colorectal cancer

**DOI:** 10.1007/s00384-023-04322-y

**Published:** 2023-02-17

**Authors:** Henna Karjalainen, Päivi Sirniö, Anne Tuomisto, Markus J. Mäkinen, Juha P. Väyrynen

**Affiliations:** 1 Cancer and Translational Medicine Research Unit, University of Oulu, POB 5000, 90014 Oulu, Finland; 2 Department of Pathology, Medical Research Center Oulu, Oulu University Hospital, Oulu, Finland

**Keywords:** Colorectal cancer, B cell, Plasma cell, T cell, Prognosis

## Abstract

**Purpose:**

The purpose of this study was to compare a B cell/plasma cell–based scoring system to T cell score and evaluate their prognostic value in colorectal cancer.

**Methods:**

We used immunohistochemistry to analyze the expression of CD20, CD138, CD3, and CD8 in 221 colorectal cancer patients. CD20+ B cell and CD138+ plasma cell densities in the tumor center and invasive margin were calculated and converted into a B cell/plasma cell score. T cell score was defined similarly, using CD3+ and CD8+ T cell densities. Their associations with tumor and patient characteristics and survival were analyzed.

**Results:**

Kaplan–Meier analysis showed a high B cell/plasma cell score was associated with a tendency towards longer survival (*p* = 0.089), but no statistically significant association was found. High T cell score associated with longer cancer-specific survival in Kaplan–Meier analysis and multivariable Cox regression analysis (*p* < 0.001). Additionally, high T cell score associated with lower disease stage (*p* < 0.001) and lesser lymphovascular invasion (*p* = 0.020).

**Conclusions:**

High T cell score is associated with longer survival and clinicopathological factors typical to less aggressive tumors. This study did not support the additional prognostic value of B cell/plasma cell quantification.

**Supplementary Information:**

The online version contains supplementary material available at 10.1007/s00384-023-04322-y.

## Introduction

Colorectal cancer (CRC) is the third most common cancer worldwide and the second most common cause of cancer deaths [1]. Prognostic classification of CRC is primarily based on the disease stage. However, tumors within the same stage may have various clinical outcomes depending on individual characteristics of the patient and disease [2–5]. Therefore, new prognostic tools are needed alongside the TNM staging system. These include molecular and genetic factors, such as microsatellite instability (MSI), *BRAF* and *KRAS* mutations, and tumor morphological factors, such as differentiation and lymphovascular invasion [3, 4, 6]. Accumulating evidence also shows the immune infiltrate has prognostic value [3, 6–8]. Precise knowledge of how different immune cell types modify tumor growth and metastasis could not only help define prognosis more accurately but also find patients for whom adjuvant chemotherapy would be most useful [3, 9, 10].

During the past decades, the tumor immune environment has been extensively studied, and it has been shown that the intra-tumoral immune cell composition and the strength of the adaptive immune reaction affect the clinical outcome [3, 7, 8, 10]. Especially, T cells have been connected to favorable prognosis, and scoring systems based on T cell densities have been developed and proposed to add on to TNM staging [10, 11]. Humoral immunity in relation to tumor progression has not been as thoroughly researched, but, according to a few studies, it seems that CD20+ (MS4A1+) B cells are associated with better prognosis [7, 8]. However, the role of B cells may be conflicted since they also seem to have some tumor-promoting qualities [12]. A few studies on plasma cells have also revealed associations with better prognosis [8, 13].

The prognostic value of CD8+ and CD3+ T cells has been utilized in development of scoring systems helping to better define prognosis in colorectal cancer [9, 10]. The Immunoscore^®^ has been internationally validated, and it seems to add on to the prognostic value of TNM staging [10]. Since the Immunoscore^®^ seems to be reproducible between observers, estimate the recurrence risk with reasonable accuracy, and help targeting adjuvant therapy to right patients, it has been proposed to be used in clinical decision-making alongside TNM staging [10]. However, it is not known, if other immune cell scoring systems could complement the prognostic information provided by the Immunoscore^®^.

The aim of this study was to compare a B cell- and plasma cell-based immune cell scoring system (based on CD20+ B cell and CD138+ (SDC1+) plasma cell densities in the tumor center and invasive margin) to the T cell score (based on CD3+ and CD8+ T cell densities in the tumor center and invasive margin, following the main principles of the Immunoscore^®^) and explore their prognostic value in colorectal cancer. Since B cells and plasma cells have previously been shown to be associated with better prognosis, a combined B-cell/plasma cell density score was hypothesized to represent a potential favorable prognostic factor that could be a supplementary tool alongside T cell score, since it has been shown that B cells and T cells work cooperatively in the anti-tumoral immune response [7].

## Materials and methods

### Patients

This study included newly diagnosed colorectal cancer patients from Oulu University Hospital between years 2006–2014, who signed an informed consent. The follow-up data was up to 120 months and was collected from clinical records and Statistics Finland. Cancer-specific survival (CSS) was defined as time from the operation to death from the same cancer, and overall survival (OS) was defined as time from operation to death of any cause. Clinical data, such as tumor location, distant metastases, and preoperative treatments, were collected from clinical records. Many patients with cT3 or cT4 rectal tumors received preoperative radiotherapy or chemoradiotherapy, and these cases were excluded from analyses due to potential influences of preoperative treatments on tumor morphology and immune cell infiltrates [14]. Cases were also excluded if there was not sufficient tumor material for immunohistochemistry or samples were not of adequate quality for the analysis of four immunohistochemistry markers for B cells, plasma cells and T cells.

### Immunohistochemistry and image analysis

We utilized immunohistochemistry to identify B cells (CD20), plasma cells (CD138), T cells (CD3), and cytotoxic T cells (CD8). B cell marker CD20 is expressed from pre-B cells to mature B cells. Plasma cell marker CD138 has reactivity also in tumor cells. CD3 is pan-T cell marker and CD8 labels cytotoxic T cells. The immunohistochemistry protocols and image analysis for T cells has been described in earlier studies [15, 16].

For CD20 and CD138 immunohistochemistry, sections of 3.5 µm from paraffin-embedded specimens were deparaffinized in xylene and rehydrated through graded alcohols. We used previously built tissue microarrays that included one to four cores of 3.0-mm diameter for each tumor [15, 16]. Sections were rinsed in distilled water and treated with Tris–EDTA buffer, pH 9.0, in a microwave oven at 800 W for 2 min and at 150 W for 15 min for antigen retrieval. Endogenous peroxidase activity was neutralized in peroxidase blocking solution (Dako S2023) for 5 min. For CD20, sections were incubated at room temperature with anti-CD20 antibody (clone L26, Dako M0755) at a dilution of 1:250 (Dako S2022) for 30 min. For CD138, sections were incubated at room temperature with anti-CD138 antibody (clone MI15, Thermo Scientific MS-1793) diluted 1:50 (Dako S2022) for 30 min. Then, both were incubated with Envision-polymer (Dako K5007) for 30 min. For both CD20 and CD138, diaminobenzidine was used as the chromogen and hematoxylin as the counterstain.

**Fig. 1:**
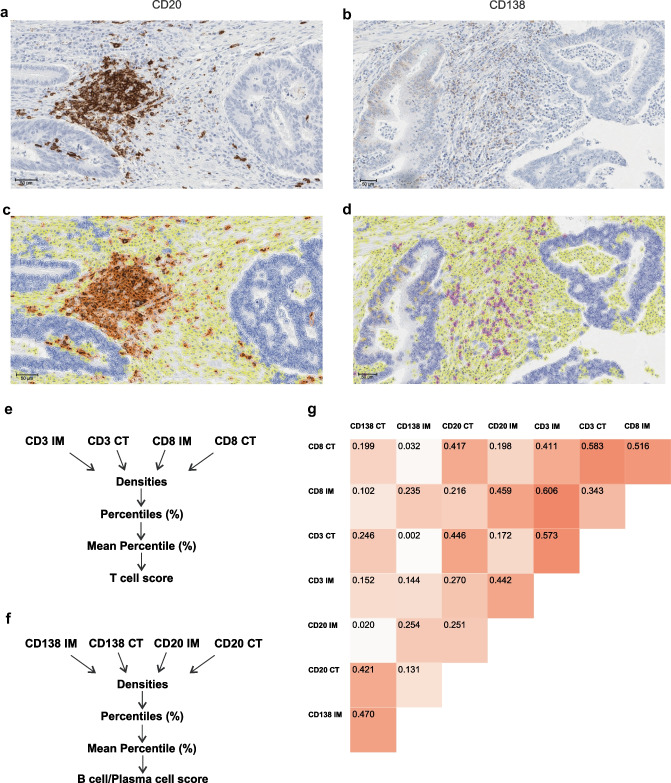
B cell, plasma cell, and T cell densities in the colorectal cancer microenvironment. Examples of CD20 and CD138 immunohistochemistry (**a–b**), and the cell types distinguished (**c–d**). Blue denotes tumor cells, brown B cells, magenta plasma cells, and yellow other cells. **e, f** Principle for calculating T cell score and B cell/plasma cell score. The correlogram shows Spearman correlations for interrelationships between different cell types (**g**). Abbreviations: CT, center of tumor; IM, invasive margin

For CD20 and CD138, the scanned images were analyzed with QuPath (Fig. 1a–d) [17]. Tissue microarray cores were annotated with the *TMA dearrayer* tool, and those unrepresentative of the tumor were excluded. The *Create thresholder* function was used to separate tissue from background. The settings for automatic *Cell detection* were optimized in small annotations, after which the detection script was run for all annotations. The *Add intensity features* function was used to calculate Haralick’s texture features, helping the subsequent cell classification, and the *Add smoothed features* function was used to calculate smoothed features. Examples of B cells/plasma cells, tumor cells, and other cells were annotated to train an object classifier to differentiate between these cell types using the *Train object classifier* function and the random forests algorithm. After successfully training the algorithm, it was used to process all tissue microarray images. Finally, areal densities of B cells and plasma cells were exported per each tissue microarray core, and averages of individual cores within each region were used as final values for subsequent analyses.

To calculate T cell score, CD3+ and CD8+ T cell densities were converted to percentiles, following the principle of the Immunoscore^®^ (Fig. 1e). The mean of the four percentile values was calculated and divided into three ordinal groups (low, intermediate, high). For the B cell/plasma cell score, following a similar procedure as for T cell score, CD20+ B cell and CD138+ plasma cell densities in the tumor center and invasive margin were converted into percentiles (Fig. 1f). The mean of the percentiles was calculated and divided into three equal sized groups, with 74 cases in the low and intermediate group and 73 in the high group.

The immunohistochemistry protocol for MMR enzymes [18] and BRAF V600E mutated protein [19] have been described in earlier studies.

### Statistical analyses

All statistical analysis were conducted using IBM SPSS Statistics for Windows (Version 27. IBM Corp). The associations between immune cell scores and clinicopathological factors were analyzed with the ${\chi }^{2}$ test. The interrelationships between different cell types were analyzed by Spearman’s rank correlation test. As our main analysis, we evaluated mortality hazard ratios (and their 95% confidence intervals) using univariable and multivariable Cox regression. Multivariable models included the following pre-determined covariates (with the reference category listed first): sex (male, female), age (< 65, 65–75, and > 75), stage (I–II, III and IV), MMR enzyme status (MMR proficient, MMR deficient), *BRAF* mutation status (wild-type, mutant), tumor location (proximal colon, distal colon, and rectum), and grade (1–2 and 3). Kaplan–Meier method was used to visualize survival curves, and the statistical significance was defined by log-rank test. All *p* values were two-tailed, and those under 0.05 were considered statistically significant.

## Results

Both T cell score and B cell/plasma cell score were successfully calculated for 221 cases, of which two patients were excluded from survival analyses because of surgery-related mortality within 30 days from surgery. Spearman correlation analyses indicated that CD3+ and CD8+ T cell densities, CD20+ B cell densities, and CD138+ plasma cell densities were mostly positively correlated (Fig. 1g). The strongest correlation was found between CD3+ and CD8+ T cells on the invasive margin (*R* = 0.606). In the invasive margin, CD20+ B cells correlated with CD8+ (*R* = 0.459) and CD3+ (*R* = 0.442) T cells. For plasma cells, the strongest correlations were found in the tumor center with CD20+ B cells (*R* = 0.421) and CD3+ T cells (*R* = 0.246).

The associations between the T cell score and tumor and patient characteristics are presented in Table 1. High T cell score was associated with lower disease stage (*p* < 0.001), higher grade (*p* = 0.037), lesser lymphovascular invasion (*p* = 0.020), and mutant *BRAF* status (*p* = 0.003). The B cell/plasma cell score did not have significant association with any of the studied clinicopathological factors, including age, sex, tumor location, stage, grade, lymphovascular invasion, MMR status, and *BRAF* status (*p* > 0.05) (Table 2).

**Table 1:** Characteristics of colorectal cancer patients according to the T cell score

		T cell score			
Characteristic	Total *N*	Low	Intermediate	High	*P*
All cases	221 (100%)	74 (34%)	74 (34%)	73 (33%)	
Sex					0.90
Female	110 (50%)	38 (51%)	38 (51%)	35 (48%)	
Male	111 (50%)	36 (49%)	36 (49%)	38 (52%)	
Age (years)					0.56
< 65	68 (31%)	28 (38%)	20 (27%)	20 (27%)	
65–75	71 (32%)	23 (31%)	25 (34%)	23 (32%)	
> 75	82 (37%)	23 (31%)	29 (39%)	30 (41%)	
Tumor location					0.31
Proximal colon	93 (42%)	27 (37%)	31 (42%)	35 (48%)	
Distal colon	60 (27%)	26 (35%)	20 (27%)	14 (19%)	
Rectum	68 (31%)	21 (28%)	23 (31%)	24 (33%)	
AJCC disease stage					< 0.001
I	45 (21%)	11 (15%)	14 (19%)	20 (28%)	
II	74 (34%)	17 (23%)	31 (42%)	26 (36%)	
III	67 (31%)	22 (30%)	24 (32%)	21 (29%)	
IV	34 (16%)	24 (32%)	5 (7%)	5 (7%)	
Tumor grade					0.037
Low grade (well to moderately differentiated)	195 (89%)	67 (92%)	69 (93%)	59 (81%)	
High grade (poorly differentiated)	25 (11%)	6 (8%)	5 (7%)	14 (19%)	
Lymphovascular invasion					0.020
No	110 (50%)	28 (38%)	37 (51%)	45 (62%)	
Yes	109 (50%)	45 (62%)	36 (49%)	28 (38%)	
MMR status					0.16
MMR proficient	191 (87%)	68 (93%)	62 (84%)	61 (84%)	
MMR deficient	29 (13%)	5 (7%)	12 (16%)	12 (16%)	
*BRAF* status					0.003
Wild-type	200 (91%)	73 (99%)	67 (91%)	60 (82%)	
Mutant	21 (9%)	1 (1%)	7 (10%)	13 (18%)	

*AJCC* American Joint Committee on Cancer, *MMR* mismatch repair

**Table 2:** Characteristics of colorectal cancer patients according to the B cell/plasma cell score

		B cell/plasma cell score			
Characteristic	Total *N*	Low	Intermediate	High	*P*
All cases	221 (100%)	74 (34%)	74 (34%)	73 (33%)	
Sex					0.93
Female	110 (50%)	37 (50%)	36 (49%)	38 (52%)	
Male	111 (50%)	37 (50%)	38 (51%)	35 (48%)	
Age (years)					0.75
< 65	68 (31%)	26 (35%)	21 (28%)	21 (29%)	
65–75	71 (32%)	21 (28%)	23 (31%)	27 (37%)	
> 75	82 (37%)	27 (37%)	30 (41%)	25 (34%)	
Tumor location					0.14
Proximal colon	93 (42%)	31 (42%)	33 (45%)	29 (40%)	
Distal colon	60 (27%)	27 (37%)	16 (22%)	17 (23%)	
Rectum	68 (31%)	16 (22%)	25 (34%)	27 (37%)	
AJCC disease stage					0.25
I	45 (21%)	12 (16%)	14 (19%)	19 (26%)	
II	74 (34%)	23 (32%)	24 (32%)	27 (37%)	
III	67 (31%)	21 (29%)	25 (34%)	21 (29%)	
IV	34 (16%)	17 (23%)	11 (15%)	6 (8%)	
Tumor grade					0.31
Low grade (well to moderately differentiated)	195 (89%)	65 (88%)	68 (93%)	62 (85%)	
High grade (poorly differentiated)	25 (11%)	9 (12%)	5 (7%)	11 (15%)	
Lymphovascular invasion					0.47
No	110 (50%)	35 (47%)	34 (47%)	41 (56%)	
Yes	109 (50%)	39 (53%)	38 (53%)	32 (44%)	
MMR status					0.88
MMR proficient	191 (87%)	65 (88%)	62 (85%)	64 (88%)	
MMR deficient	29 (13%)	9 (12%)	11 (15%)	9 (12%)	
*BRAF* status					0.61
Wild-type	200 (91%)	68 (92%)	68 (92%)	64 (88%)	
Mutant	21 (20%)	6 (8%)	6 (8%)	9 (12%)	

*AJCC* American Joint Committee on Cancer, *MMR* mismatch repair

**Fig. 2:**
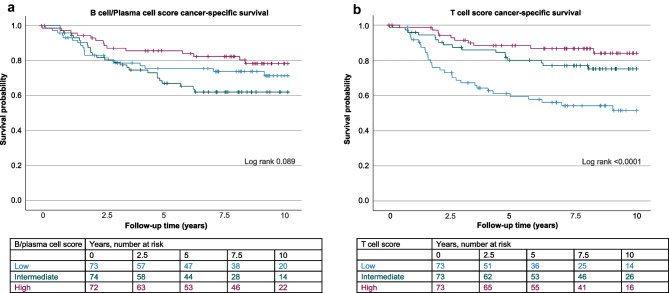
Kaplan–Meier estimates for cancer-specific survival. Kaplan–Meier curves for **a** B cell/plasma cell score and **b** T cell score

In survival analysis, there were 104 deaths, including 59 cancer deaths, and the median follow-up time was 118 months (IQR 100–120) within censored cases. High T cell score was associated with prolonged cancer-specific survival in the Kaplan–Meier analysis (*p* < 0.0001; Fig. 2b), while high B cell/plasma cell score associated with a tendency towards longer cancer-specific survival (*p* = 0.089; Fig. 2a). In multivariable Cox regression analysis that included both lymphocyte scores along with selected clinicopathologic factors, such as disease stage and MMR status, high T cell score independently associated with longer cancer-specific survival (HR 0.22 compared to low T cell score; 95% CI 0.09–0.50; *p*_trend_ < 0.001) and overall survival (HR 0.44 compared to low T cell score; 95% CI 0.25–0.75; *p*_trend_ = 0.002), while B cell/plasma cell score did not significantly associate with cancer-specific survival (*p*_trend_ = 0.30) or overall survival (*p*_trend_ = 0.25) (Table 3, Table S1).

**Table 3:** Univariable and multivariable Cox regression models for cancer-specific survival and overall survival according to T cell score and B/plasma cell score

		Colorectal cancer-specific survival			Overall survival		
	No. of cases	No. of events	Univariable HR (95% CI)	Multivariable HR (95% CI)	No. of events	Univariable HR (95% CI)	Multivariable HR (95% CI)
B/plasma cell score							
Low	73	19	1 (referent)	1 (referent)	31	1 (referent)	1 (referent)
Intermediate	74	26	1.38 (0.77–2.50)	1.71 (0.89–3.32)	44	1.50 (0.94–2.37)	1.71 (1.03–2.83)
High	72	14	0.68 (0.34–1.35)	1.41 (0.64–3.07)	29	0.85 (0.51–1.42)	1.33 (0.76–2.34)
* P*_trend_			0.29	0.30		0.53	0.25
T cell score							
Low	73	32	1 (referent)	1 (referent)	46	1 (referent)	1 (referent)
Intermediate	73	17	0.42 (0.24–0.76)	0.53 (0.27–1.02)	30	0.49 (0.31–0.78)	0.49 (0.30–0.81)
High	73	10	0.25 (0.12–0.51)	0.22 (0.09–0.50)	28	0.47 (0.30–0.76)	0.44 (0.25–0.75)
* P*_trend_			< 0.001	< 0.001		0.001	0.002

Multivariable Cox proportional hazards regression models were adjusted for age (< 65, 65–75, > 75), sex (male, female), stage (I–II, III, IV), mismatch repair (MMR) enzyme status (MMR proficient, MMR deficient), *BRAF* mutation status (wild-type, mutant), tumor location (proximal colon, distal colon, rectum), B cell score (low, intermediate, high), T cell score (low, intermediate, high), and grade (1–2, 3)

*CI* confidence interval, *HR* hazard ratio

To gain further insights into the role of the components of T cells score and B cell/plasma cell score, we analyzed their prognostic impact as separate variables (Table 4). Multivariable Cox regression analysis showed that high CD3+ T cell density in the tumor invasive margin had the strongest prognostic impact and was independently associated with longer cancer-specific survival (HR for high vs. low 0.33; CI 0.16–0.69; *p*_trend_ = 0.001), and high CD3+ T cell density (*p*_trend_ = 0.025) and high CD8+ T cell density (*p*_trend_ = 0.042) in the tumor center also associated with longer cancer-specific survival in multivariable Cox regression analysis. Of the components of B cell/plasma cell score, high CD20+ B cell densities in the tumor center showed a tendency towards association with longer cancer-specific survival in the univariable analysis (*p*_trend_ = 0.065), while no statistically significant associations were observed in multivariable Cox regression models (*p*_trend_ > 0.25).

**Table 4:** Univariable and multivariable Cox regression models for cancer-specific survival and overall survival according to CD138+ plasma cell, CD20+ B cell, CD3+ T cell, and CD8+ T cell densities

		Colorectal cancer-specific survival			Overall survival		
	No. of cases	No. of events	Univariable HR (95% CI)	Multivariable HR (95% CI)	No. of events	Univariable HR (95% CI)	Multivariable HR (95% CI)
CD138+ plasma cell density (tumor center)							
Low	73	15	1 (referent)	1 (referent)	30	1 (referent)	1 (referent)
Intermediate	74	28	1.94 (1.04–3.64)	1.29 (0.67–2.48)	43	1.53 (0.96–2.43)	1.43 (0.88–2.33)
High	72	16	0.99 (0.49–2.00)	0.82 (0.39–1.72)	31	0.96 (0.58–1.59)	0.81 (0.48–1.36)
* P*_trend_			0.94	0.91		0.85	0.42
CD138+ plasma cell density (invasive margin)							
Low	73	19	1 (referent)	1 (referent)	36	1 (referent)	1 (referent)
Intermediate	73	20	0.96 (0.51–1.80)	0.48 (0.24–0.96)	32	0.81 (0.50–1.31)	0.57 (0.34–0.95)
High	73	20	1.03 (0.55–1.94)	1.26 (0.66–2.44)	36	0.98 (0.62–1.55)	1.09 (0.67–1.78)
* P*_trend_			0.92	0.56		0.93	0.81
CD20+ B cell density (tumor center)							
Low	73	24	1 (referent)	1 (referent)	39	1 (referent)	1 (referent)
Intermediate	74	20	0.74 (0.41–1.35)	0.87 (0.46–1.64)	33	1.45 (0.91–2.31)	0.86 (0.53–1.39)
High	72	15	0.55 (0.29–1.04)	0.64 (0.31–1.36)	32	1.07 (0.66–1.74)	0.81 (0.49–1.35)
* P*_trend_			0.065	0.25		0.12	0.42
CD20+ B cell density (invasive margin)							
Low	73	21	1 (referent)	1 (referent)	39	1 (referent)	1 (referent)
Intermediate	73	24	1.06 (0.59–1.90)	1.51 (0.81–2.82)	35	0.84 (0.53–1.33)	0.12 (0.69–1.82)
High	73	14	0.59 (0.30–1.16)	1.20 (0.56–2.55)	30	0.68 (0.42–1.10)	1.20 (0.71–2.03)
P_trend_			0.14	0.51		0.12	0.49
CD3+ T cell density (tumor center)							
Low	71	29	1 (referent)	1 (referent)	39	1 (referent)	1 (referent)
Intermediate	75	15	0.40 (0.22–0.75)	0.46 (0.24–0.87)	31	0.62 (0.39–0.99)	0.63 (0.39–1.02)
High	73	15	0.43 (0.23–0.79)	0.48 (0.23–1.00)	34	0.71 (0.45–1.13)	0.63 (0.38–1.06)
* P*_trend_			0.004	0.025		0.15	0.076
CD3+ T cell density (invasive margin)							
Low	72	35	1 (referent)	1 (referent)	45	1 (referent)	1 (referent)
Intermediate	73	14	0.33 (0.18–0.61)	0.47 (0.24–0.91)	32	0.56 (0.36–0.89)	0.74 (0.45–1.21)
High	74	10	0.22 (0.11–0.44)	0.33 (0.16–0.69)	27	0.43 (0.27–0.69)	0.52 (0.31–0.88)
* P*_trend_			< 0.001	0.001		< 0.001	0.014
CD8+ T cell density (tumor center)							
Low	73	28	1 (referent)	1 (referent)	41	1 (referent)	1 (referent)
Intermediate	73	15	0.74 (0.25–0.89)	0.62 (0.33–1.18)	31	0.65 (0.41–1.04)	0.73 (0.45–1.18)
High	73	16	0.51 (0.28–0.95)	0.50 (0.24–1.04)	32	0.69 (0.44–1.10)	0.67 (0.40–1.11)
* P*_trend_			0.024	0.042		0.11	0.10
CD8+ T cell density (invasive margin)							
Low	73	27	1 (referent)	1 (referent)	42	1 (referent)	1 (referent)
Intermediate	73	19	0.68 (0.38–1.22)	0.83 (0.44–1.55)	35	0.80 (0.51–1.25)	0.83 (0.51–1.34)
High	73	13	0.41 (0.21–0.80)	0.48 (0.23–0.99)	27	0.56 (0.34–0.90)	0.57 (0.33–0.98)
* P*_trend_			0.008	0.051		0.02	0.042

Multivariable Cox proportional hazards regression models were adjusted for age (< 65, 65–75, > 75), sex (male, female), stage (I–II, III, IV), mismatch repair (MMR) enzyme status (MMR proficient, MMR deficient), *BRAF* mutation status (wild-type, mutant), tumor location (proximal colon, distal colon, rectum), and grade (1–2, 3)

*CI* confidence interval, *HR* hazard ratio

## Discussion

Our goal was to compare a B cell/plasma cell-based scoring system in colorectal cancer with the previously well-established T cell score and explore their prognostic significance and associations with clinicopathological factors. Our results highlight the strong association of high T cell score with longer survival, but do not support additional value of B cell/plasma cell density quantification.

We found that a high T cell score was associated with lower stage and lesser lymphovascular invasion, which suggests that high T cell score is associated with a less aggressive tumor phenotype. Similar results were shown before by Ko and Pyo [20]. High densities of CD3+ and CD8+ T cells have been associated with tumors showing less markers of early metastatic invasion [21]. It seems that high T cell score/density is associated with lesser metastasis and lower stage. However, high T cell score also associated with high grade and *BRAF* mutations, which are linked to more aggressive tumors. *BRAF* mutation is an independent prognostic factor for poor survival in colorectal cancer [6]; however, Cen et al. showed that, in *BRAF* mutated tumors, there was more immune cell infiltration, including cytotoxic T cells, which is in line with our results [22]. On the contrary, although T cell and B cell densities were moderately correlated, we did not find any statistically significant associations between the B cell/plasma cell score and clinicopathological factors, suggesting that T cell densities are more closely associated with other tumor characteristics.

The significance of humoral immunity in colorectal cancer is relatively understudied compared to the significance of cell-mediated immunity, and, to our knowledge, a B cell/plasma cell based prognostic scoring system had not been previously tested. Our results suggest that higher densities of CD20+ B cells in the tumor center tend to be associated with longer cancer-specific survival (*p* = 0.065), but no statistically significant survival associations were found for either B cell or plasma cell densities. A reasonable explanation for this could be the fact that the number of B cells in the tumor is low compared to T cells which makes their definition more prone to error. Additionally, the impact of humoral immunity might be partially mediated through anti-tumor antibodies which can be produced from afar [23], and therefore the B cell or plasma cell count in the tumor itself is not that significant. CD138 immunohistochemistry was not easy to quantify, due to variable staining in tumor epithelium, which may have led to imprecise plasma cell densities in some tumors. IGKC may represent a more ideal marker for plasma cells compared to CD138, since it exclusively stains plasma cells/plasma blasts and is therefore easier to interpret [24]. Another possibility would be CD20-CD79A double-stain, where CD79A+CD20- cells would constitute plasma cells [25]. It is possible that using CD138 staining in our study contributed to the suboptimal prognostic value. In subsequent studies, it is also relevant to analyze the significance of various B cell and plasma cell subpopulations since these cells may also comprise some immunosuppressive cell types such as regulatory B cells.

Our results on the T cell score were in line with previous studies since we also found a strong association between high T cell score and longer survival [9, 10, 26]. In an international validation cohort by Pagès et al., it was proven that in patients with stages I–III colorectal cancer, those with high Immunoscore had longest survival and lowest risk of recurrence [10]. It has been stated that, compared to the TNM classification, the Immunoscore could more accurately define prognosis, predict tumors of high recurrence risk, and identify patients who would most benefit from adjuvant therapies [10]. Immunoscore has also been associated with lower number of metastases [27]. Our study supports the prognostic impact of T cell score independent of age, gender, stage, grade, MSI, *BRAF* status, tumor location, and the B cell score.

Previously, it has been described that the prognostic impact of B cells was connected to simultaneous high count of T cells and was lost if T cell density was low, regardless of the B cell density [7]. It is likely that at least part of the prognostic significance of B cells is mediated through a cooperative function with T cells [28]. The cooperative function of CD20+ B cells and CD8+ T cells has been described also in ovarian cancer, where the contribution of B cells was mainly by antigen-presenting function and not by producing anti-tumoral antibodies [29]. We analyzed the correlations between CD20+ and CD8+ cell densities in the tumor center and invasive margin, which were fairly strong, supporting the idea that there may be cooperative anti-tumoral function between B and T cells.

There were a few limitations to this study. First, the study population was quite small, which may limit the accuracy of the results. The CD138 staining was rather difficult to interpret because it has reactivity in both tumor and plasma cells, which may affect the accuracy of the plasma cell count. Also, tissue microarray cores cover only a small fraction of the tissue and may not be fully representative of the whole section. However, the tissue microarrays that were used in this study were based on large cores of 3.0-mm diameter, increasing the area subjected to analysis. Moreover, tissue microarrays enabled us to stain large numbers of tumors with high consistency. Our cohort was analyzed for two key molecular features of colorectal cancer, mismatch repair deficiency, and *BRAF* status, which enabled us to study the prognostic significance of immune cells independent of these factors. Nevertheless, larger studies comparing the prognostic significance of B cells and T cells are warranted.

In conclusion, high T cell score is associated with less aggressive tumors and better prognosis, which makes it a useful prognostic tool, while our study does not support the additional prognostic significance of B cell/plasma cell density scoring. In future research, it would be important to understand the role of different B cell subsets in tumor progression and anti-tumor immunity.

### Supplementary Information

Below is the link to the electronic supplementary material.

Supplementary file1 (PDF 243 KB)

## Data Availability

The datasets generated and/or analyzed during this study are not publicly available. The sharing of data will require approval from the relevant ethics committee and/or biobank. Further information including the procedures to obtain and access data of Finnish Biobanks are described at https://finbb.fi/en/fingenious-service.

## Abbreviations

HR: Hazard ratio
OS: Overall survival
MMR: Mismatch repair
IGKC: Immunoglobulin kappa C
MSI: Microsatellite instability
