# Supplementary material for: A prognostic score based on B cell and plasma cell densities compared to T cell densities in colorectal cancer
Source: Int J Colorectal Dis. 2023 Feb 17;38(1):47. doi: 10.1007/s00384-023-04322-y (PMC9938036; doi:10.1007/s00384-023-04322-y)
Supplement: Supplementary file 1 — Supplementary file1 (PDF 243 KB) [file 384_2023_4322_MOESM1_ESM.pdf]

A prognostic score based on B cell and plasma cell densities compared to T cell densities in colorectal cancer

Henna Karjalainen[1], Päivi Sirniö[1,2], Anne Tuomisto[1], Markus J. Mäkinen[1,2], Juha P. Väyrynen[1,2]

[1]Cancer and Translational Medicine Research Unit, University of Oulu, Oulu, Finland

[2]Department of Pathology, Medical Research Center Oulu, Oulu University Hospital, Oulu, Finland

Supplementary material

Table S1. Multivariable Cox regression models for cancer-specific survival and overall survival according to all variables

Variable	Cancer-specific survival	Overall survival
	Multivariable HR (95% CI)	Multivariable HR (95% CI)
B/plasma cell score		
Low	1 (referent)	1 (referent)
Intermediate	1.77 (0.92-3.41)	1.71 (1.03-2.83)
High	1.48 (0.68-3.25)	1.33 (0.76-2.34)
T cell score		
Low	1 (referent)	1 (referent)
Intermediate	0.56 (0.29-1.08)	0.49 (0.30-0.81)
High	0.22 (0.09-0.52)	0.44 (0.25-0.75)
Age		
<65	1 (referent)	1 (referent)
65-75	3.24 (1.54-6.82)	3.10 (1.63-5.88)
>75	3.71 (1.75-7.89)	6.43 (3.53-11.70)
Sex		
Male	1 (referent)	1 (referent)
Female	0.88 (0.50-1.56)	0.86 (0.60-1.30)
Tumor location		
Proximal colon	1 (referent)	1 (referent)
Distal colon	1.73 (0.85-3.52)	1.28 (0.73-2.25)
Rectum	1.29 (0.65-2.58)	1.36 (0.81-2.27)
Disease stage		
I-II	1 (referent)	1 (referent)
III	3.86 (1.71-8.70)	1.38 (0.84-2.27)
IV	31.22 (13.35-73.05)	9.74 (5.52-17.19)
Tumor grade		
Low-grade (well to moderately differentiated)	1 (referent)	1 (referent)
High-grade (poorly differentiated)	2.47 (1.11-5.50)	1.28 (0.67-2.46)
MMR status		
MMR proficient	*	1 (referent)
MMR deficient		1.09 (0.48-2.47)
BRAF mutation		
Negative	1 (referent)	1 (referent)
Positive	2.02 (0.44-9.20)	1.37 (0.54-3.50)

Abbreviations: CI, confidence interval; HR, hazard ratio; MMR, mismatch repair.
* Not included in the colorectal cancer-specific mortality model because of no events in the MMR deficient group.
